# Making Data Reports Useful: From Descriptive to Predictive

**DOI:** 10.7759/cureus.10920

**Published:** 2020-10-12

**Authors:** Alfred C Ma

**Affiliations:** 1 Medicine, Mansfield International College, Fullerton, USA; 2 Anesthesiology, Riverside University Health System Medical Center, Moreno Valley, USA

**Keywords:** data and analytics, machine leaning, decision-support tools

## Abstract

The purpose of analyzing data is to transform it into useful knowledge. Descriptive analytics renders factual information about research and events that can be used to relate an organization’s environment to its activities. However, descriptive analytics alone is not enough to gain understanding and possibly predict the future. Minding only the output of such an analysis can mislead the researcher and decisionmaker. Because many factors influence results, it is essential to advance the prediction of future challenges through statistical analytics and factual patterns that dictate the environment with scientifically tested models. The data patterns, types of analysis, and attributes the prediction will be based on are all important. Data influenced by unforeseen variables make for poor predictions, such as the evening capacity report data in this study.

## Introduction

Exploratory research helps scientists develop initial hunches or insights [1]. However, the tradition in medical science research presentations is descriptive analytics. Descriptive analytics and statistics provide images of what happened in experiments based on past data. Yet, for health care organizations, this reactive approach helps to present what happened historically, but it does not help forecast future outcomes. The key to descriptive analytics is to present data effectively with statistical functions to reflect what really happened and with visual tiles for easy understanding.

Predictive analytics, if used appropriately, has been proven to foresee the future with precision [2]. This study continues to examine emergency department (ED) capacity reporting data during the coronavirus disease 2019 (COVID-19) pandemic the author descriptively examined in a previous publication in this journal (August 2020) that described the importance of presenting descriptive data in a meaningful, continuous, and easy-to-understand fashion [3]. From data to insights, the study further advocates for predictive analytics, which provides evidence of the associations between variables and identifies areas in need of improvement. The aim is to help health care organizations to utilize available data analytics tools to produce valuable information applicable to assist health care leaders in decision making and improve services in the future. Application of predictive analytics may not yield completely accurate results, but it does provide a clearer picture of what might happen in the future based on past events.

## Materials and methods

As presented in a previous report [3], the hospital studied here is a state safety net health organization. It is a 440-bed acute care teaching hospital in the Western region of the United States. It has a 45-bed capacity emergency room and is licensed for 48 intensive care beds.

Twice a day, the nursing house supervisor distributes the emergency room capacity alert reports to medical staff via email. These reports include ED census and numbers of ED holds with and without bed assignment. It also includes COVID-19 cases held in the ED and, through calculation, bed availability is determined. Thus, there are five parameters identified in this study: ED census, ED hold with bed assignment, ED hold without bed assignment, COVID-19 patients holding in ED without bed, and bed availability at the hospital.

Predictive analytics involves forecasting, which dictates timely decision making. In this study, forecasting was applied to predict bed availability at the hospital through ED capacity reporting data. It is not possible to predict the future demand for hospital beds with certainty, but an estimate can be realized from past data. The data collected in this study revealed trends from May 2020 to July 2020. Linear regression performed by Excel® (Microsoft, Redmond, WA, USA) was used to determine attributes’ associations and forecast bed availability with suitable independent variables. A paired t-test was applied to test the significant similarity between forecasted outputs and what really happened during the month of August 2020 (the true data). Waikato Environment for Knowledge Analysis (WEKA®, University of Waikato, New Zealand) machine learning software was used to visualize the forecasting results and to test the degree of accuracy of the models by classification.

## Results

Data in this study consisted of the morning reported data, which represented how resources were allocated at night, and the evening reported data, which represented how efficiently resource allocation occurred during the day.

**Figure 1:**
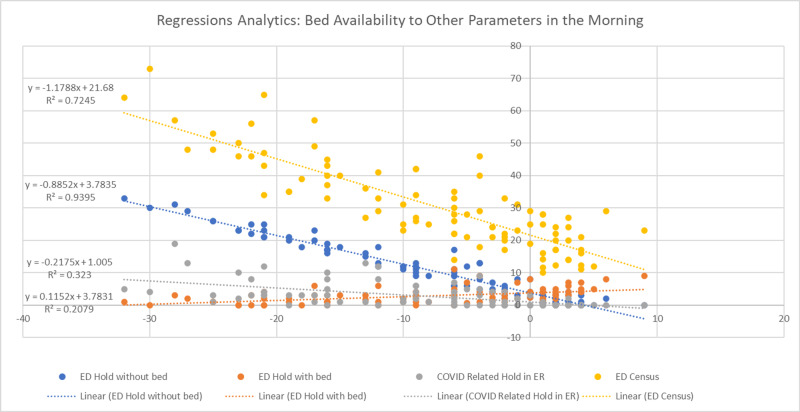
Regression analysis on bed availability to other parameters in the morning COVID-19: coronavirus disease 2019

The analysis began with regression analysis and forecasting. Regression is about quantifying the relationship between two or more variables. As presented in Figure 1, the study began with regression analytics to determine what ED holding situations (dependent variables) would be at differing levels of bed availability (the independent variable). On the horizontal axis is bed availability at the hospital, and on the vertical axis is the bed availability related to ED census, ED holding without bed assignment, ED holding with bed assignment, and COVID-19 related hold in ED. The regression plots then tried to fit a straight line to the data and put a formal equation (y) into each of the dependent variables.

The graph’s equations connect bed availability at the hospital to each holding parameters and an R-square value (how good the regression line is). In general, an R-square value above 70% can be depicted as a well-modeled regression able to capture the majority of value points. For example, regression between bed availability at the hospital and ED hold without bed assignment is considered a particularly good fit with an R-square value of 0.9395 (93.95%). Yet, lower values of R-square output, such as that of ED hold with bed (20.79%) and COVID-19-related hold in ED (32.30%), should not be immediately discounted where the P-value of the parameter in computing summary may represent some merits in the regression model. In some cases, it is possible that additional predictors can increase the true explanatory power of the model. Even when R-square is low, low P-values still indicate a real association between the significant predictors and the response variable.

Multiple linear regression estimates the relationship between a quantitative dependent variable and two or more independent variables using a straight line. In multiple regression analytics, multiple independent predictors are used in an attempt to forecast bed availability at the hospital (the response variable). The preliminary multiple regression examination revealed that both ED hold with bed (P-value = 0.3231) and COVID-19-related hold in the ED (P-value = 0.2795) were not significantly associated with bed availability at the hospital and were omitted from the analysis. The final model was designed to associate two predictors, ED census and ED hold without bed assignment, to bed availability at the hospital and were deemed to be strongly reliable. The R-square values of the final multiple regression models in the morning and evening were 95% and 83%, respectively.

The final regression model output summary is shown in Table 1. It depicted the calculated values presented by multiple regression of the final model in the morning and evening. P values were such that the null hypothesis was rejected with 95% level of confidence in all predictors. All evidence available strongly validated the data model.

**Table 1:** Multiple regression model outputs in the morning and the evening.

Report		Coefficients	Standard Error	t Stat	P-value
Morning Report	Intercept	1.469913571	0.79189844	1.856189502	0.066735491
	ED Census	0.129510082	0.04328516	2.992020377	0.003585531
	ED Hold without bed	-1.239970731	0.065639774	-18.89053923	6.79269E-33
Evening Report	Intercept	-1.686498927	1.150544857	-1.465826314	0.146219823
	ED Census	0.145442066	0.023451589	6.201800011	1.71448E-08
	ED Hold without bed	-1.178215425	0.059385233	-19.84020869	1.97733E-34

**Figure 2:**
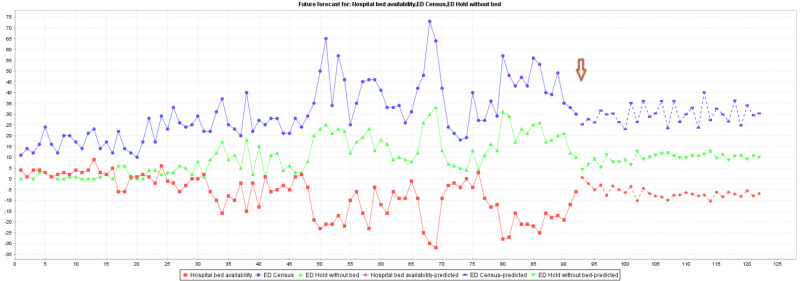
WEKA® forecasting output of morning dataset (resource allocation at night)

**Figure 3:**
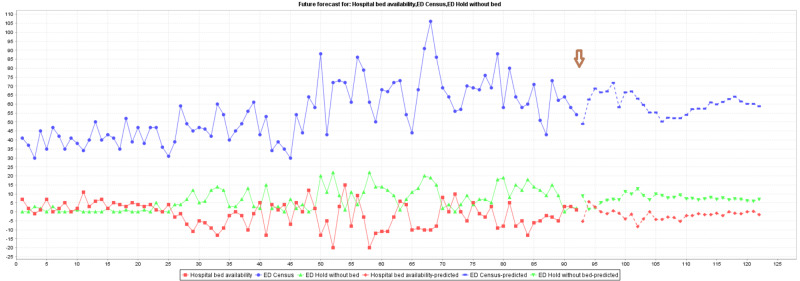
WEKA® forecasting output of evening dataset (resource allocation during the day)

Visualization of forecasting exercises was completed with WEKA® machine learning software. The forecasting results are shown in Figure 2 and Figure 3. Figure 2 depicts forecasting results based on the morning dataset, and Figure 3 presents the forecasting output using the evening dataset. The forecast setting was placed to forecast 30 data points to mime 30 days in August 2020 and displayed after the brown arrow in the figures.

It is important to verify and test whether the forecasting output is factually accurate or close enough to be significantly similar to what really happened in the month of August 2020. To examine the performance of the forecasting model, paired T-tests of two sample means were used to compare the forecast 30 data points with the factual data collection in August of 2020 (Table 2).

**Table 2:** T-Test, Paired Two Sample for Means on predicted and August values

	AM		PM	
	Predicted	August 2020	Predicted	August 2020
ED Census	29.7±4.5	30.7±8.7	59.6±5.8	56.3±13.9
ED Hold Without Bed	9.5±2.1	7.7±6.1	7.5±2.4	3.8±3.2 *
Bed Availability	-6.3±2.5	-4.7±6.6	-1.2±2.4	0.7±3.1 *
Mean±SD * P-value <0.05				

There was no statistically significant difference between all pairs, namely the ED census, ED hold without bed assignment, and the availability of bed at the hospital with the morning data set. Yet, the comparison of bed availability and ED hold without bed assignment showed a significant difference between the predicted value and the data collected in August 2020 in the evening data set. The significant difference between forecast and real events during the day can be explained by the unexpected variabilities of factors that can influence bed availability at the hospital. Allocation of resources, human and bed, is much easier to achieve during the day and, in turn, created too many possible variations to predict. The prediction exercise was then further scrutinized using machine learning classification for confirmation.

Machine learning classification is a form of data analysis used to build models describing the accuracy of classes the data represent [4]. Here, the study sought to examine how well the algorism predicts correctly when bed availability is positive or negative. Given the regression analysis output, the new task was to test if the regression analysis was compatible through machine learning classification. Using machine learning models to examine a proposed strategy or decision-making process through historical data is vital [5]. There are many analytics models available in machine learning to help management categorically predict the executability of a decision through accuracy values based on past experiences.

The datasets were uploaded into WEKA® machine learning software to test diagnostic decision-making accuracy from regression and forecasting results. Before classification of test data was performed, supervised learning using Support Vector Machine classification with 70% random split ratio was chosen to test the accuracy of model-classified instances. Accuracy of classified instances refers to the percentage of training data the algorism gets correct. The classification returned a 96.63% accuracy, resulting in confidence in the fitness of the model.

The classification tests of the datasets were established using the forecasted 30 value points and what actually happened in August 2020. Table 3 depicts the results from both morning and evening reports. Accuracy was in the 90th percentile in the morning and evening reports’ predicted outputs. Factual (what really happened in August) outputs in the evening dataset were less fitted, with lower accuracy percentage (equal or less than 80%), which concurs with the results of the paired T-test (Table 2).

**Table 3:** Support Vector Machine classification to test predicted and factual output categorical accuracy

Report	Tested data	Accuracy	Precision	Recall	F-Measure
Morning Report	Predicted	100%	100%	100%	100%
	August 2020	89.67%	91.30%	88.70%	90.10%
Evening Report	Predicted	96.67%	97.00%	96.70%	96.70%
	August 2020	80.00%	80.60%	80.00%	78.40%

Model performance in machine learning is estimated in terms of its accuracy in predicting the given or training data based on unknown or test data. An accuracy metric is used to measure the algorithm’s performance and is calculated in the form of a percentage. It is the measure of how accurate the model's prediction is compared to the true data. Accuracy is used along with precision and recall, which are other metrics that use various ratios. These metrics provide insight into how the algorithm classifies data points based on prediction correctness.

## Discussion

It is necessary to bear in mind the hospital is constantly trying to allocate resources during high census, which may distort the forecast in predictive models and other tests. In regression analysis, the coefficient of determination represents the mean change in the response for one unit of change in the predictor. Low R-square values are problematic when prediction needs to be precise. Here, the ED census alone is not enough to reflect bed availability at the hospital, and the additional support of using ED hold without bed assignment can better predict it.

It is predictively valid to forecast bed availability at the hospital with both ED census and ED hold without bed assignment as predictors. The limitation is obvious in applying evening ED capacity report data to predict bed availability, as these data were influenced by the effectiveness of stakeholders’ allocating hospital resources during the day. Resource allocation is often carried out after morning clinical activities are over and can take place too late in the day to make the evening report useful. Thus, it is critical and meaningful for the nursing house supervisor to distribute ED capacity information to the medical staff in the early morning and alert stakeholders to provide beds and other resources effectively as early as possible during the day. ED census alone does not well reflect bed availability. It is the combination of two predictors, ED census and ED hold without bed assignment, that enable stakeholders to reflect the scarcity of resources with confidence.

## Conclusions

Valuable administrative data in health care organizations is severely underutilized, and the lack of available research in health care operational data is supporting such claims. Data analytics are proven to be helpful in forecasting and decision making. There is much data collection in health care, and the content is not limited to clinical and evidence-based scientific research and learning. It also includes daily administrative and business data, which is mostly idle in database storage. Keeping health care business intelligence in silos limits the improvement of health delivery and health quality as well as cost reduction. The changing landscape of healthcare creates great demand for health data analytics. Data-driven business intelligence and analytics enhance healthcare performance, revenues, and patient experience. Today, technologies like predictive analytics and machine learning are readily available to transform health care business, strategy, and communications.
